# SPINE20 recommendations 2021: spine care for people’s health and prosperity

**DOI:** 10.1007/s00586-022-07194-y

**Published:** 2022-04-07

**Authors:** Giuseppe Costanzo, Bernardo Misaggi, Luca Ricciardi, Sami I. AlEissa, Koji Tamai, Fahad Alhelal, Yahya Alqahtani, Hana I. Alsobayel, Markus Arand, Massimo Balsano, Thomas R. Blattert, Marco Brayda-Bruno, Jamiu O. Busari, Marco Campello, Harvinder S. Chhabra, Francesco Ciro Tamburrelli, Pierre Côté, Bambang Darwono, Frank Kandziora, Giovanni A. La Maida, Eric J. Muehlbauer, Raghava D. Mulukutla, Paulo Pereira, Shanmuganathan Rajasekaran, Dominique A. Rothenfluh, William J. Sullivan, Eeric Truumees, Edward J. Dohring, Tim Pigott, Ajoy P. Shetty, Marco G. A. Teli, Jeffrey C. Wang, Christopher Ames, Johannes R. Anema, Anand Bang, Kenneth M. C. Cheung, Douglas P. Gross, Scott Haldeman, Salvatore Minisola, Rajani Mullerpatan, Stefano Negrini, Louis-Rachid Salmi, M. Silvia Spinelli, Adriaan Vlok, Kwadwo P. Yankey, Fabio Zaina, Ahmed Alturkistany, Jörg Franke, Ulf R. Liljenqvist, Michael Piccirillo, Margareta Nordin

**Affiliations:** 1 Sapienza Rome University, Rome, Italy; 2 Orthopaedic Institute Gaetano Pini-CTO, Milan, Italy; 3 National Guard Health Affairs, Riyadh, Saudi Arabia; 4 Department of Orthopedics, Graduate School of Medicine, Osaka Metropolitan University, Osaka, Japan; 5 National Guard Hospital, Riyadh, Saudi Arabia; 6 Presidency of State Security, Riyadh, Saudi Arabia; 7 King Saud University, Riyadh, Saudi Arabia; 8 Medical Faculty, University of Ulm, Ulm, Baden-Württemberg Germany; 9 University and Hospital Trust of Verona, Verona, Italy; 10 Schwarzach Orthopaedic Clinic, Schwarzach, Germany; 11 IRCCS Orthopedic Institute Galeazzi, Milano, Italy; 12 Faculty of Health, Medicine and Life Sciences, Maastricht University, Maastricht, The Netherlands; 13 New York University Grossman School of Medicine, New York, NY USA; 14 Indian Spinal Injuries Center, New Delhi, India; 15 Orthopedic Catholic University, Rome, Italy; 16 Ontario Tech University, Oshawa, ON Canada; 17 Gading Pluit Hospital, North Jakarta, Jakarta, Indonesia; 18 Center for Spinal Surgery and Neurotraumatology, Frankfurt, Germany; 19 Spine Surgery Department, Orthopedic Institute Gaetano Pini, Milan, Italy; 20 North American Spine Society, Chicago, USA; 21 Udaiomni Hospital, Hyderabad, India; 22 University of Porto, Porto, Portugal; 23 Ganga Hospital, Coimbatore, India; 24 Oxford University Hospitals NHS Foundation Trust, Oxford, UK; 25 Vanderbilt University Medical Center, Nashville, USA; 26 University of Texas, Austin, USA; 27 Spine Institute of Arizona, Scottsdale, AZ USA; 28 EUROSPINE, Zurich, Switzerland; 29 Walton Centre NHS Trust, Liverpool, UK; 30 University of Southern California Spine Center, Los Angeles, CA USA; 31 University of California San Francisco, San Francisco, CA USA; 32 Amsterdam University Medical Center, Amsterdam, The Netherlands; 33 Society for Education, Action and Research in Community Health, Gadchiroli, Maharashtra India; 34 The University of Hong Kong, Hong Kong, China; 35 University of Alberta, Edmonton, Canada; 36 University of California, Irvine, CA USA; 37 Sapienza University of Rome, Rome, Italy; 38 Mahatma Gandhi Mission Institute of Health Sciences, Navi Mumbai, India; 39 University La Statale, Milan, Italy; 40 IRCCS Istituto Ortopedico Galeazzi, Milan, Italy; 41 Université de Bordeaux, Bordeaux, France; 42 Gaetano Pini Institute, Milano, Italy; 43 Stellenbosch University, Cape Town, South Africa; 44 FOCOS Orthopaedic Hospital, Accra, Ghana; 45 ISICO (Italian Scientific Spine Institute), Milan, Italy; 46 King Faisal Specialist Hospital and Research Center, Jeddah, Saudi Arabia; 47 Klinikum Magdeburg gGmbH, Magdeburg, Germany; 48 St. Franziskus-Hospital, Münster, Germany; 49 New York University, New York, NY USA

**Keywords:** SPINE20, G20, Recommendation, Spine, Advocacy group

## Abstract

**Purpose:**

The focus of SPINE20 is to develop evidence-based policy recommendations for the G20 countries to work with governments to reduce the burden of spine disease, and disability.

**Methods:**

On September 17–18, 2021, SPINE20 held its annual meeting in Rome, Italy. Prior to the meeting, the SPINE20 created six proposed recommendations. These recommendations were uploaded to the SPINE20 website 10 days before the meeting and opened to the public for comments. The recommendations were discussed at the meeting allowing the participants to object and provide comments.

**Results:**

In total, 27 societies endorsed the following recommendations. SPINE20 calls upon the G20 countries: (1) to expand telehealth for the access to spine care, especially in light of the current situation with COVID-19. (2) To adopt value-based interprofessional spine care as an approach to improve patient outcomes and reduce disability. (3) To facilitate access and invest in the development of a competent rehabilitation workforce to reduce the burden of disability related to spine disorders. (4) To adopt a strategy to promote daily physical activity and exercises among the elderly population to maintain an active and independent life with a healthy spine, particularly after COVID-19 pandemic. (5) To engage in capacity building with emerging countries and underserved communities for the benefit of spine patients. (6) To promote strategies to transfer evidence-based advances into patient benefit through effective implementation processes.

**Conclusions:**

SPINE20’s initiatives will make governments and decision makers aware of efforts to reduce needless suffering from disabling spine pain through education that can be instituted across the globe.

## Introduction of SPINE20

Global demographic and health changes have led to a rapid increase in the number of people experiencing disability due to non-communicable diseases (NCDs) [1–3]. Musculoskeletal disorders are both predominant and the leading cause of disability within the NCDs group; this translates to 1.71 billion people affected, equating to 149 million years lived with disability [3]. Among all musculoskeletal disorders, spine pain is the leading cause of disability with more than half a billion individuals0 worldwide experiencing disability due to low back pain [1]. In 2017, the World Health Organization (WHO) launched the Rehabilitation 2030 initiative to mobilize the global community and reduce the burden of disability [4].

Recently in 2019, four large spine care and research non-governmental organizations (EUROSPINE, the North American Spine Society, the German Spine Society, and the Saudi Spine Society) formed SPINE20, an advocacy group to bring global attention to spine disorders (Table 1). In 2020, the Italian Spine Society (SICV and GIS), the Indonesian Spine Society (ISS), and the Association of Spine Surgeons of India (ASSI) joined, which were the dominant societies of future G20 host countries. The main focus of SPINE20 is to develop evidence-based policy recommendations for the G20 countries to work with governments to reduce the burden of spine disease, disability, and injuries.

**Table 1:** The SPINE20 suggested multi-dimensional initiatives

Establish high educational standards for spine care providers that ensure quality care throughout the world
Invest in spine research that increases our knowledge to improve spine care globally
Adopt spine disability prevention strategies that lead to healthier populations
Improve the ability to address issues relative to the aging population with spine disorders through government policies and recommendations

### How can SPINE20 inform G20 nations?

The report from the May 21, 2021, G20 Global Health Summit summarized its support of these efforts in its statement: “We, the leaders of G20 and other states, in the presence of the heads of international and regional organizations meeting at the Global Health Summit in Rome, May 21, 2021, having shared our experience of the ongoing global COVID-19 pandemic, and welcoming relevant work in this regard.” The G20 Global Health Summit provided 16 recommendations related to the COVID pandemic and recognized the very damaging impact of the pandemic on progress toward achieving the Sustainable Development Goals (SDG). In this document, the G20 group reaffirmed their commitment to achieving the goals to strengthen efforts to build back better (as in UNGA resolution, September 11, 2020) [5], and to the International Health Regulations 2005, which together will improve resilience and global health outcomes. [6]

As a newly formed advocacy group, SPINE20 is committed to working to facilitate the implementation of these recommendations. Specifically, SPINE20 recognizes the COVID-19 pandemic has led to a reduction in the availability and access of non-COVID-related surgical and rehabilitation care. Moreover, SPINE20 recognize that the burden of spine disorders in general, and low back pain in particular, will likely increase due to unmet rehabilitation needs and the growing number of people with “Long-COVID.”[7] This is particularly important because spine problems are the greatest contributor to disability and health expenditures globally. [8] Furthermore, low back pain affects most severely female, elderly, and low-income populations. [9] The SPINE20 recommendations to the G20 group are meant to highlight deficiencies in prevention, education, access to spine care, and mitigate disability from spinal disorders in an effort to reduce the wave of disability that may follow the COVID-19 pandemic.

The recommendations are intended to benefit individuals with spine and low back ailments, thereby benefitting the community and ultimately the country adopting the recommendations. The world has different needs depending on the geographical location and socioeconomic status. One recommendation will not fit all. The potential solutions and strategic plans must be developed by the local health ministry with support from spine societies, public health officials, the communities, and the available care systems. The recommendations must be adapted and accepted by the cultural environment, have a positive human and economic impact, and finally must show progress over time.

### SPINE20 domain concept

The concept indicates that all recommendations should be based on the specific domain that SPINE20 considered as critical for global improvement in spine health. In 2020, SPINE20’s first recommendations were created based on 11 domains including “Spinal disability,” “Prevention,” “Value-based care,” “Patient’s safety,” “Access to care,” “Education,” “Research and innovation,” “Pediatric,” “Aging spine,” “Spinal cord injury,” and “Low back pain.” In 2021, the Publication and Recommendation Committee decided to retain 5 domains from 2020 to 2021 including “Spinal disability,” “Value-based care,” “Access to care,” “Research and innovation,” and “Aging spine.” Additionally, this year the committee has decided to add two critical domains: “Implementation and outcomes” and “Building capacity.” There is a need to implement global outcomes to measure progress and to create and monitor benchmarks to improving spine care for governments. Building capacity means to create a sustainable environment to reduce disability and enhance spine health.

### Recommendations and rationale proposed for SPINE20 2021

**Fig. 1:**
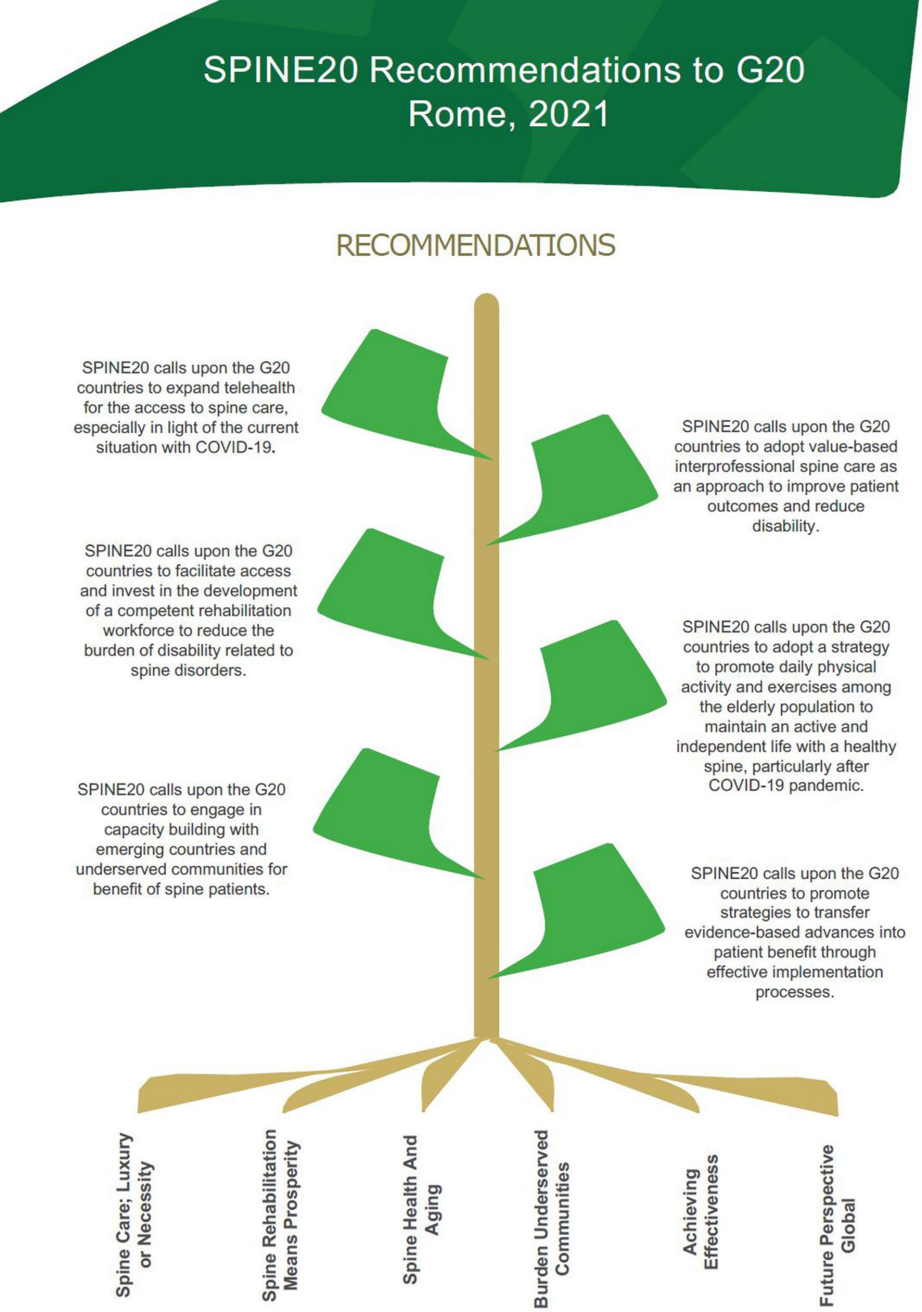
SPINE20 Recommendations to G20 countries, 2021

On September 17 and 18, 2021, SPINE20 held its second annual meeting in Rome, Italy, with the theme “Spine Care for People’s Health and Prosperity.” Before the meeting, the SPINE20 Scientific Committee and Publication and Recommendation Committee created six proposed Recommendations including their rationale. These recommendations were uploaded to the SPINE20 website (https://spine20.org/event/) 10 days before the second annual meeting and opened to the public for comments. Subsequently, the recommendations were discussed at the hybrid annual meeting September 18, 2021, allowing the participants to object and provide comments. Finally, 188 participants from 34 societies have approved with no objection the proposed SPINE20 recommendation statement for 2021. The recommendation paper, which are listed below with their respective rationale, was again reviewed and endorsed by 27 societies (Table 2). Recommendations acted upon by SPINE20 participating societies will be tracked and presented at the upcoming SPINE20 meeting in Bali, Indonesia, (August 4–5, 2022) where the next G20 summit will take place. The following recommendations from the SPINE20 gathering in 2021were endorsed (Fig. 1).

**Table 2:** The list of societies endorsed the SPINE20 2021 recommendations (December 19, 2021)

Society	Nations	SPINE20 recommendations
Asian spinal cord network	India	Under reviewing
Asociación Mexicana de Cirujanos de Columna	Mexico	Under reviewing
Association of spinal surgeons of Russia	Russia	Reviewed and endorsed
Association of spine surgeons of India	India	Reviewed and endorsed
Associazione dei Cavalieri Italiani del Sovrano Militare Ordine di Malta	Italy	Reviewed but not endorsed
Australian pain society	Australia	Reviewed and endorsed
Brazil spine society	Brazil	Reviewed and endorsed
Egyptian spine association	Egypt	Reviewed and endorsed
Egyptian spine study group	Egypt	Reviewed and endorsed
EUROSPINE	International	Reviewed and endorsed
Foundation of orthopedics and complex spine	Ghana	Under reviewing
German spine society	Germany	Reviewed and endorsed
Hellenic society spine surgeons	Greece	Reviewed and endorsed
Indonesia spine society	Indonesia	Reviewed and endorsed
International society on scoliosis orthopedic and rehabilitation treatment	International	Reviewed and endorsed
Inter-state council secretariat	India	Reviewed and endorsed
Italian society of physical and rehabilitation medicine	Italy	Under reviewing
Italian spine society (SICV and GIS)	Italy	Reviewed and endorsed
Japanese society for spine surgery and related research	Japan	Reviewed and endorsed
Middle East spine society	International	Reviewed and endorsed
North America spine society	USA	Reviewed and endorsed
Order of Malta	Italy	Under reviewing
Saudi association of neurological surgery	Saudi Arabia	Reviewed and endorsed
Saudi physical therapy association	Saudi Arabia	Reviewed and endorsed
Saudi spine society	Saudi Arabia	Reviewed and endorsed
Sociedad Iberolatinoamericana de Columna	International	Reviewed and endorsed
Società Italiana di Ortopedia e Traumatologia	Italy	Reviewed and endorsed
Society for education, action and research in community health	India	Reviewed and endorsed
Society indian physiotherpy	India	Reviewed and endorsed
South african spine society	South Africa	Reviewed and endorsed
Spinal cord society	India	Reviewed and endorsed
Ukrainian spine society	Ukraine	Under reviewing
World federation of chiropractic	International	Reviewed and endorsed
World spine care	International	Reviewed and endorsed

### Recommendation: research and innovation, access to care

### SPINE20 calls upon the G20 countries to expand telehealth for the access to spine care, especially in light of the current situation with COVID-19

#### Background

The COVID-19 pandemic has greatly affected life, health, and society worldwide [10]. Though the emergent countermeasures, including home confinement and lockdowns, were effective to some extent in preventing the spread of COVID-19 in the community, the prolongation of these countermeasures is also negatively affecting many aspects of people’s lives, such as overall decreasing physical activity and daily exercise [11]. In addition, due to the pandemic, patients with spinal disorders are experiencing delays in timely access to needed care.

#### Problem

Many patients with spinal disease have lost access to care, especially during the COVID-19 pandemic.

#### Potential solutions

SPINE20 calls upon the G20 countries to expand telehealth for spine care, especially due to the current ongoing situation with COVID-19 [12]. By taking this measure, patients with spinal diseases can obtain timely advice toward alleviating pain and recognizing critical symptoms that need urgent care, and thus obtaining treatment in a timely manner [13]. Telehealth has not been widely adopted in the diagnosis and treatment of spine disease. This might reflect the inherent limitations of telehealth visits for performing physical examinations and demonstrating rehabilitation instructions, but also reflects the previous restrictions on billing for application of electronic health and mobile health, telehealth visits, and the need for technical troubleshooting during telehealth visits [14, 15]. Although before COVID there was a lack of any strong impetus to change our approach to patient care, there is now a critical ongoing need to provide alternative means of evaluating and treating patients affected by the pandemic. Hence, SPINE20 recommend that each G20 country urgently develop a system to support telehealth in this COVID-19 era in order to ensure access to care for patients with spinal diseases. We believe that economic evaluations of telehealth, including the resulting increased productivity of workers worldwide, will validate the benefits of telehealth spine care [16].

### Recommendation: value-based care

### SPINE20 calls upon the G20 countries to adopt value-based interprofessional spine care as an approach to improve patient outcomes and reduce disability

#### Background

Lower back pain affects 80–90% of the adult population in both industrialized and emerging countries. Evidence suggests that 30% of affected individuals experience some disability and loss of function [3]. Prolonged pain leads to loss of function, loss of ability to work, poverty, and the loss of the ability to provide for oneself, one’s family, and the community, and is often a primary cause for loss of quality-of-life, ability to function, and independent activity of daily living [17].

#### Problem

Due to its high prevalence and chronicity, spinal disease remains a leading cause of disability.

#### Potential solutions

SPINE20 calls upon the G20 countries to understand that effectiveness in spine care occurs when different specialists work together, i.e., interprofessional care, using evidence-based methods to prescribe the most effective and efficient care for any given patient. By utilizing the varied education and experience of professionals from different specialties, patients are the ultimate benefactors [18]. Spine Care providers include primary care physicians, surgeons, physiatrists, physical therapists, occupational physicians/therapists/nurses, chiropractors, pain management specialists, psychologists, psychiatrists, social workers, orthotists, assistive technologists, vocational counselors, peer counselors, and others who can all contribute key insights that will optimize comprehensive care for patients [19]. Governments should encourage interprofessional care by providing incentives or fair payment paradigms that bring multiple perspectives to bear on any given patient.

### Recommendation: spinal disability, access to care

### SPINE20 calls upon the G20 countries to facilitate access and invest in the development of a competent rehabilitation workforce to reduce the burden of disability related to spine disorders

#### Background

The COVID-19 pandemic has led to an unprecedented increase in unmet rehabilitation needs. Moreover, the prevalence of chronic spine pain and disability is likely to increase because of the growth in the number of individuals with “Long COVID” in which spine and bodily pain are frequently reported symptoms. Even if COVID is resolved, low back and neck pain will continue to be the main causes of disability globally and this puts a great burden on the health care systems and economic welfare of our societies [8]. However, most people who would benefit from rehabilitation for their back and neck pain cannot access these services because they are not available, are not affordable, or because of a shortage of health care providers in their community [3].

#### Problem

Despite significant health expenditures, the global burden of disability related to spine pain continues to grow and the COVID-19 pandemic has amplified this problem [20].

#### Potential solutions

SPINE20 calls upon the G20 countries to facilitate and invest in the development of a competent rehabilitation workforce to reduce the burden of disability related to spine ailments. The health, well-being, and productivity of the population will benefit from: (1) Developing a rehabilitation workforce that can deliver high quality and value-based rehabilitation for people with spine pain; (2) Promoting the delivery of high quality and value-based rehabilitation for people with spine pain; and (3) Facilitating easy access to high quality and value-based rehabilitation aimed at returning injured workers to a productive life. The government efforts should target the timely delivery of quality rehabilitation to these individuals to promote population prosperity. The delivery and accessibility of rehabilitation services (including return-to-work interventions) that are supported by high-quality evidence must be prioritized.

### Recommendation: aging spine

### SPINE20 calls upon the G20 countries to adopt a strategy to promote daily physical activity and exercises among the elderly population to maintain an active and independent life with a healthy spine, particularly after COVID-19 pandemic

#### Background

The degenerative spinal diseases occurring later in adult life have a significant impact on lifestyle expectations and activities of the elderly population [21]. In addition, osteoporotic vertebral fractures are major problems for the elderly population [22]. These conditions effect the role of the elderly in their family, and also effect local economic productivity.

#### Problem

The social and financial burden placed upon local communities and on the G20 countries by spinal degenerative diseases and osteoporotic vertebral fractures in the elderly is significantly increasing.

#### Potential solutions

SPINE20 calls upon the G20 countries to adopt strategies to promote daily exercises among the elderly population to maintain an active and independent life with a healthy spine. Physical activity and exercises, proper nutrition, and a smoking-free lifestyle are proven to reduce the severity of osteoporosis and frailty of the trunk among the elderly, helping them to maintain the ability to ambulate and carry out daily activities [23]. Education for health care practitioners on prevention strategies for the aging population along with the concurrent education of the elderly population are the best tools to prevent degenerative changes and osteoporotic fractures that often lead to severe symptoms and disability [24]. Finally, a proper interprofessional treatment strategy for patients with osteoporosis and/or degenerative spinal diseases should be defined according to scientific evidence-based assessments, and these approaches must be subject to human and financial long-term outcome and cost analyses [25].

### Recommendation: capacity building

### SPINE20 calls upon the G20 countries to engage in capacity building with emerging countries and underserved communities for benefit of spine patients

#### Background

Spinal problems are among the most frequent causes of loss of function and disability [17]. It is one of the major causes of significant loss of quality-of-life, especially in the underserved areas of the world. Ignorance, cultural and financial problems, and geographical and political impediments are essential factors that limit people’s access to care and these factors deserve to be considered and discussed in detail [26]. It is crucial to understand the role of inadequate information and lack of awareness of the general public in preventing spinal diseases and spinal injuries [27, 28].

#### Problem

There is a poor standard of spine care in different parts of the world, especially in underserved communities or in emerging countries.

#### Potential solutions

SPINE20 calls upon the G20 countries to increase spine care capacity-building in emerging countries and in all underserved communities [29]. To address the disproportionate distribution and access to optimal spine care, senior administrators and policymakers in developing and underserved countries need to network and create alliances with G20 countries, and to adopt specific strategies based on the best clinical practices of countries with robust spine health care systems [30]. Long-term, realistic, region-specific goals for the improvement in spine care need to be developed based on region-specific factors such as the local epidemiology, availability of resources, social beliefs, attitudes/mindset, and urban versus rural distribution of health services. Strategies to improve the standards of spine care also need to focus on cost-effective high-impact practices, locally relevant clinical guidelines, professional oversight, targeted education/professional training, organizational change, and dedicated research. More proactive collaboration and support from the G20 scientific communities, individually or in working groups, is needed to help build and promote autonomous local scientific bodies in underserved communities.

### Recommendation: implementation and outcomes

### SPINE20 calls upon the G20 countries to promote strategies to transfer evidence-based advances into patient benefit through effective implementation processes

#### Background

Proving the effectiveness of a clinical approach is not always enough to guarantee its adoption by health services and health care professionals. It has always been a challenge to find the best way to enhance the incorporation of evidence-based practices and thereby increase their public health impact. The lack of a common language, agreement on transformative goals, an embedded evaluation process, common plans, and a shared agenda, in addition to the mentality of ‘short-termism’, are some of the main obstacles to translating evidence-based scientific discoveries into widespread patient benefit [31].

#### Problem

It is reported that evidence-based practices take an average of 17 years to be incorporated into routine general practice in health care, even though research constantly produces confirmed findings that can contribute to more effective and efficient health care. [32]

#### Potential solutions

SPINE20 calls upon the G20 countries to promote strategies that enhance the translation of the evidence-based discoveries into patient benefit at a global level through effective implementation processes. Addressing the gaps between knowledge and practice with efficient strategies should be a policy priority. [33, 34] A range of strategies is available to overcome these gaps. Stakeholder engagement, effectiveness studies, research synthesis, artificial intelligence, and mathematical modeling are some of the methods used by implementation scientists to identify strategies that embed evidence-based interventions into clinical practice and public health programs. While there is insufficient evidence to adequately support the use of some guideline implementation strategies, such as traditional educational strategies and guideline dissemination in isolation, there is convincing evidence in favor of the use of multifaceted interventions, interactive education, and clinical reminder systems for the effective implementation of clinical guidelines. [33] Furthermore, one of the most important aspects of the implementation process is economic evaluation. It is a crucial tool that always needs to be incorporated into the implementation decision-making process for any adopted strategy because it has been found to be the predominant reason (or “excuse”) that change is not implemented. We need to further promote research on the costs and cost–benefit analyses of guideline implementation strategies, along with the other environmental, organization, and individual clinician factors that are associated with effective implementation strategies [33].

## Conclusions

SPINE20 is created as an advocacy group for spine societies around the globe, and for governments, institutions, and other organizations to highlight evidence and valued-based spine care that can help prevent disability. Its recommendations are intended to reduce the burden of disease related to spinal disorders by engaging governing stakeholders in the development of evidence-based policies. SPINE20 can serve as a resource of expertise for local and/or global advisement to mitigate disability from spine ailments. It is the hope of the founding group that its advocacy will reduce needless suffering and costs from disabling pain through education about rehabilitation and other treatment modalities that can be instituted across the globe.

## Data Availability

The datasets generated during and/or analyzed during the current study are available from the corresponding author on reasonable request.
